# Overcoming the clarification challenges of high cell density culture

**DOI:** 10.1186/1753-6561-9-S9-P35

**Published:** 2015-12-14

**Authors:** Laura Gimenez, Elie E Kawkabani, Pieter Jacobs, Laetitia Malphettes

**Affiliations:** 1 Biotech Sciences, UCB Pharma, Braine l'Alleud, Belgium

## Introduction

Cell line engineering and process intensification efforts have led to high performing fed-batch cell culture processes where cell density can reach >30x106 cells/mL. These high performance processes also come with new challenges, in particular developing efficient and scalable primary recovery processes.

Centrifugation coupled with depth filtration is a standard approach for clarification of mammalian cell culture broth. However, with high cell density cultures, it can become challenging to obtain low turbidity clarified cell culture fluid (CCCF), and the filtration surface area required for clarification may become limiting when scaling up.

## Objective

We explored novel clarification strategies for cell culture processes using CHO cell lines producing monoclonal antibodies, with the goal of removing more impurities during harvest and increasing the robustness of this process step, while opening up possibilities for the reduction of the filtration surfaces.

## Determination of flocculation conditions

All the work described in this abstract was performed on CHO cell culture processes producing monoclonal antibodies (mAb). Cell density at harvest was 20-30x106 cells/ml and viability 60-70%. Turbidity of the cell culture broth at harvest was>2500NTU.

A positively charged flocculation agent, available in pharmaceutical grade and suitable for GMP manufacturing, was selected. Screening studies were performed at 2L scale in order to identify the most suitable flocculant concentration and to study the impact of agitation on the flocculation process. After flocculation, samples were spun down in a dead end centrifuge and turbidity of the supernatant was measured as an indicator of flocculation efficiency. Selected conditions were: flocculant concentration 0.0375%; agitation = 100rpm.

## Scale-up to 80L

The flocculation conditions selected at 2L scale were scaled-up to 80L scale, representative of our GMP manufacturing scale. The flocculated broth was processed through disc-stack centrifugation, depth filtration and membrane filtration. The impact on product recovery, product quality, impurity removal and filtration performance was assessed.

## Impact on filter capacity

### Depth filters

**Figure 1:**
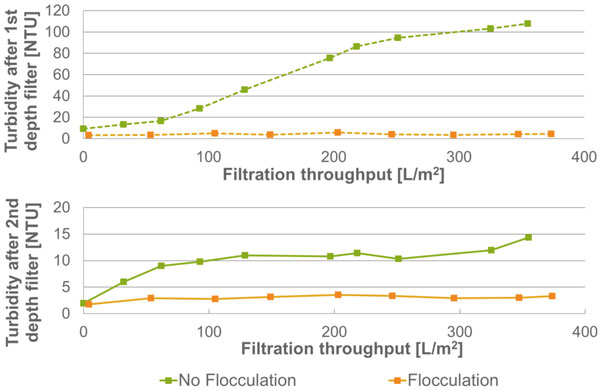
**Permeate turbidity according to filtration throughput**.

Following disc-stack centrifugation, the broth (mAb 1) was processed through a series of two depth filters. Filtration was performed at constant flow rate. Turbidity of permeate was used to monitor filtration efficiency. Results are shown in Figure 1.

### Membrane filters

The depth filtered material was then processed through bioburden reduction filters of either 0.2µm or 0.1µm cut-off. Maximum filtration throughput was used to determine filtration efficiency. Results are shown in Table 1.

**Table 1:** Impact of flocculation on filter capacity and CCCF turbidity

	Depth filter capacity [L/m^2^]	0.2µm filter capacity [L/m^2^]	0.1µm filter capacity [L/m^2^]	CCCF Turbidity [NTU]
**No flocculation**	250	1018	16	10

**Flocculation**	>350	6500	569	3

### Impact on product recovery and quality

Impact of flocculation on product recovery and quality was tested for 3 different mAbs.Titer was measured in clarified cell culture fluid by protein A HPLC. Acidic Peak Group (APG) and High Molecular Weight Species (HMWS) levels were measured on Protein A eluate.

It was shown that the addition of the flocculant did not impact product recovery and harvest yield for any of the 3 mAbs tested.

Product quality was also not impacted by the new clarification process.

### Impurity removal

Treated and untreated CCCF (mAb 1) were analyzed in order to evaluate the impact of flocculation on impurity removal prior to downstream purification.

It was shown that the introduction of the flocculant did not affect HCP content, but reduced DNA level by 90%.

## Conclusions

Optimal flocculation agent concentration and agitation conditions were selected at small scale. Flocculation was then successfully introduced into a clarification process representative of GMP manufacturing scale, including disc-stack centrifugation and depth filtration. Flocculation was shown to have no impact on product recovery and quality while enabling to increase impurity removal prior to purification, as well as filtration capacity, improving robustness and scalability of the process. Current work is focusing on studying the impact on chromatography resins and demonstrating clearance of the flocculation agent during downstream process.

